# Role of prenatal undernutrition in the expression of serotonin, dopamine and leptin receptors in adult mice: Implications of food intake

**DOI:** 10.3892/mmr.2013.1853

**Published:** 2013-12-10

**Authors:** LETICIA MANUEL-APOLINAR, LUISA ROCHA, LETICIA DAMASIO, EMILIANO TESORO-CRUZ, ARTURO ZARATE

**Affiliations:** 1 Endocrine Research Unit, National Medical Center, Mexican Social Security Institute, Mexico City, Mexico; 2 Department of Pharmacobiology, Center for Research and Advanced Studies, National Medical Center, Mexican Social Security Institute, Mexico City, Mexico; 3 Department of Research and Animal Facility, INCMN, National Medical Center, Mexican Social Security Institute, Mexico City, Mexico; 4 Immunology Research Unit, National Medical Center, Mexican Social Security Institute, Mexico City, Mexico

**Keywords:** prenatal undernutrition, food intake, obesity, leptin, serotonin, dopamine receptors

## Abstract

Perturbations in the levels of serotonin expression have a significant impact on behavior and have been implicated in the pathogenesis of several neuropsychiatric disorders including anxiety, mood and appetite. Fetal programming is a risk factor for the development of metabolic diseases during adulthood. Moreover, previous studies have shown that serotonin (5-HT), dopamine and leptin are important in energy balance. In the present study, the impact of maternal malnutrition-induced prenatal undernutrition (UN) was investigated in mice and the expression of 5-HT_1A_, dopamine (D)1, D2 and Ob-Rb receptors was analyzed in the hypothalamus during adulthood. The UN group showed a low birth weight compared with the control group. With regard to receptor expression, 5-HT_1A_ in the UN group was increased in the hypothalamus and D1 was reduced, whereas D2 showed an increase from postnatal day (P)14 in the arcuate nucleus. Ob-Rb receptor expression was increased in the hypothalamus at P14 and P90. These observations indicated that maternal caloric restriction programs a postnatal body weight gain in offspring with an increased food intake in early postnatal life which continues into adulthood. In addition, UN in mice was found to be affected by Ob-Rb, 5-HT_1A_ and D1/2 receptor expression, indicating that these observations may be associated with hyperphagia and obesity.

## Introduction

The fetal programming hypothesis suggests that diseases of the fetus originate through adaptation, which occurs with fetal undernutrition (UN). These adaptations may be vascular, metabolic or endocrinological and permanently change the function and structure of the body in adult life.

In a previous study, Barker *et al* suggested that infants with low birth weight (LBW) had an increased risk of developing obesity, hypertension and diabetes (1). Population studies and animal models have revealed critical periods when offspring are most vulnerable to environmental effects, including maternal nutritional imbalance (2,3). Thus, fetal programming is considered to be a potential mechanism contributing to the development of obesity. In rats, malnutrition during select periods of pregnancy causes LBW in newborns (4).

When newborns were fed *ad libitum*, growth-restricted offspring demonstrated accelerated growth, currently termed ‘catch-up growth’ (5), such that their body weight exceeded that of the control groups (6–8). Therefore, evidence exists that early postnatal growth acceleration, which is normally considered necessary, may exacerbate metabolic dysfunction during later life (9). Moreover, excessive food intake and subsequent obesity increase the risk of developing chronic diseases. Thus, fetal programming may modify appetite-regulating hormones and neurotransmitters in undernourished newborns (9). Findings of previous studies have revealed that serotonin (5-HT) is a neurotransmitter that also acts as an important hormone in an increasing number of physiological processes outside of the brain. Thus, serotonergic and dopaminergic receptors (D) may be targets for the treatment of cognitive deficits and feeding disorders. This vulnerability may result from abnormalities in the development and integration of serotonergic and dopaminergic projections during the prenatal period (10,11).

Therefore, the aim of the present study was to determine whether prenatal UN modified the expression of the 5-HT_1A_, D1, D2 and Ob-Rb receptors in the hypothalamus of adult mice.

## Materials and methods

### Animals

The present study utilized a model of fetal programming via maternal malnutrition, in which 50% of food was restricted during pregnancy to produce LBW in the offspring (12). The protocol was approved by the Local Animal Research Committee and was conducted in accordance with the American Association for Accreditation of Laboratory Care and National Institutes of Health guidelines. Animals were assigned to one of two nutritional groups: i) Control (C) group, fed *ad libitum* during gestation; and ii) UN group, fed with a 50% food-restricted diet during the final week of gestation. The day of birth was designated as postnatal day (P)0. Following birth, offspring were weighed and litter sizes were normalized to eight offspring per litter for adequate and standardized nutrition until weaning. For the offspring study, animals were immediately classified into either the UN group, where mothers received the restricted diet during the last gestation week or to the C group, as aforementioned. Mothers from UN and C pups were fed *ad libitum* during lactation. Each litter from the two groups was weighed weekly. The first weight was recorded at P0 and subsequent weights were taken at P7 and P14, until P90. In the period following weaning, food intake was monitored in the UN and C offspring from the post-weaning period to P90. Offspring were sacrificed at various postnatal ages by rapid decapitation.

### RNA extraction

Hypothalamic studies were performed at P0, P14 and P90. Pups were sacrificed by decapitation and brains were rapidly removed and blotted free of excess blood. Sections of the hypothalamus were removed from each group, rapidly frozen in dry ice and stored at −70°C until use for RNA extraction.

Total cellular RNA was isolated from the hypothalamic tissue using TRIzol reagent in 100 mg tissue according to the manufacturer’s instructions. Briefly, RNA was precipitated from the TRIzol solution following the addition of chloroform followed by isopropyl alcohol and then washed in 75% ethanol in diethyl pyrocarbonate-treated water (DEPC). The ethanol was then removed and the RNA pellets were air-dried prior to the addition of 20 μl RNase-free water. To remove contaminating DNA, the samples were treated with DNAase. Total RNA concentration was determined spectrophotometrically at 260/280 nm, and the isolated RNA was stored at −70°C.

### Reverse transcription-polymerase chain reaction (RT-PCR) analysis of the Ob-Rb receptor

Purified total RNA (2 μg) was used as a template to generate first-strand cDNA (Fermentas First-Strand cDNA kit; Thermo Fisher Scientific, Waltham, MA, USA) which was amplified with a specific primer for Ob-Rb receptor and tubulin using a Fermentas Pyrostart RT-PCR kit (Thermo Fisher Scientific). The PCR mixture contained *Taq* DNA polymerase and buffers (PCR amplification buffer with 30 mM MgCl), and 10 mM dNTP, and 15 μM each of the 5′ and 3′ primers against the Ob-Rb receptor were added to cDNA samples generated from the hypothalamus samples. Primers used for the Ob-Rb receptor were: Forward: 5′-CCAGGTGAGGAGCAAGAGAC-3′ and reverse: 5′-CTGCACAGTGCTTCCCACTA-3′ (product size, 470 bp); β-tubulin, forward: 5′-TCAGCGTGGTGCCCTCAC-3′ and reverse: 5′-GTGAGCTCAGGCACCGTC-3′ (product size, 370 bp). Initial denaturation at 95°C for 5 min was followed by 35 cycles of denaturation for 1 min at 95°C, annealing for 1 min at 55°C and extension for 1 min at 72°C. PCR was terminated by a final extension at 72°C for 5 min using a Mastercycler ep gradient S thermocycler (Eppendorf, Hauppauge, NY, USA). Subsequent assay results were analyzed relative to a housekeeping gene (tubulin) within the same sample to normalize for possible variations in RNA quality, quantity and efficiency. Tubulin levels were analyzed independently and did not vary in any of the experimental groups.

### Electrophoresis

The samples were separated in 2% agarose gels in the presence of ethidium bromide. The optical density (OD) of bands was measured using a Kodak Transilluminator Gel Logic 200 (Kodak, Rochester, NY, USA). Data are presented as a ratio of leptin receptor expression to tubulin.

### Autoradiography

Animals were sacrificed and whole brains were rapidly removed, blotted free of excess blood, rapidly frozen in pulverized dry ice and stored at −70°C for later use. For 5-HT_1A_, D1 and D2 receptor autoradiography, brains were sliced into coronal sections on a cryostat (CM 1510; Leica Camera AG, Solms, Germany) at −20°C, each with a section thickness of 20 μm. Sections were thaw-mounted on gelatin-coated slides and stored at −70°C in plastic bags until the day of incubation. The tissue was rehydrated at room temperature only during ligand incubation. Standard conditions were used, for example the concentration of tritium ligand (equivalent to its kDa) and the concentration of ligand for non-specific binding, temperature, incubation time and washing time.

For autoradiography studies, the incubation experiments consisted of tissue sections pre-incubated in Coplin jars at room temperature in 40 ml of a solution of Tris-HCl (pH 7.4) incubation buffers. Tissue sections were then incubated in the same buffer containing the radioligand at an adequate final concentration. Non-specific binding was generated by the addition of butaclamol (1 μM) for D1 and D2 and WAY100635 (10 μM) for 5-HT_1A_. Following incubation, the sections were washed in ice-cold (4°C) buffer solution twice for 5 min and immediately dipped into cold distilled water to remove any salts. Tissue sections were dried under a gentle stream of cool air. Slides were arrayed in X-ray cassettes together with tritium standards (Amersham Pharmacia Biotech, Piscataway, NJ, USA) and were exposed to tritium-sensitive film (Kodak hyperfilm; Eastman Kodak) at room temperature for 2 or 3 months (13–15). Films were developed and fixed at room temperature (Table I). ODs of the selected areas appeared on autoradiograms where these were determined using a video-computer enhancement program (Jandel video analysis software; Jandel Scientific, Corte Madera, CA, USA) and the OD values were transformed into receptor density values expressed as fmol/mg protein. Results were obtained from 10 animals per group. Brain areas and nuclei were identified using the Paxinos and Watson atlas.

### Statistical analysis

Data are presented as mean ± SEM. Differences between groups were considered statistically significant based on one-way ANOVA followed by a parametric multiple comparison (Tukey’s test). Statistical analyses were performed using Prism Software (Graph Pad Prism 5 for Windows, San Diego, CA, USA). P<0.05 was considered to indicate a statistically significant difference.

## Results

### Growth

**Figure 1:**
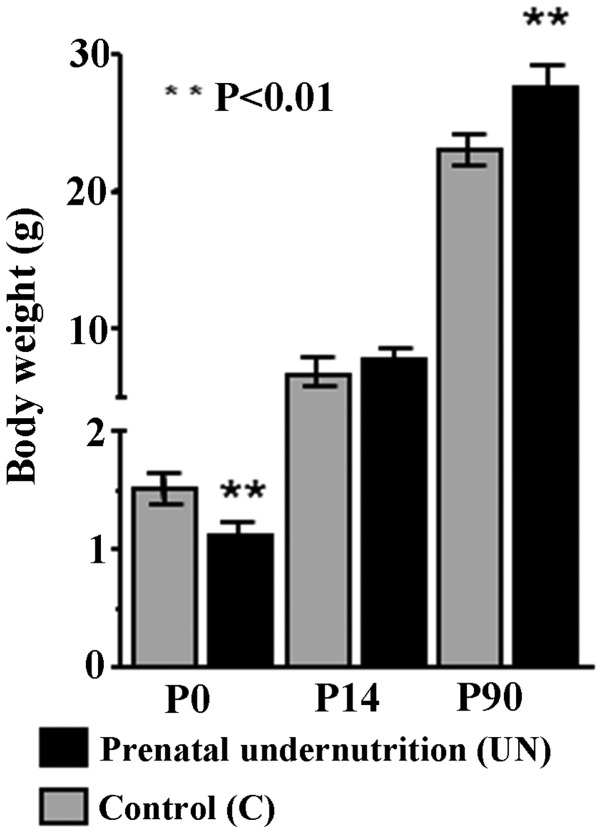
Representation of body weight in prenatal UN vs. C groups on postnatal days 0 (P0), 14 (P14) and 90 (P90). Data are expressed as mean ± SEM; n=10/group. ^**^P<0.01, vs. C. C, control; UN, undernutrition; P, postnatal.

As a result of food restriction during gestation, body weights of the UN group at birth were lower than those of the C group (1.10±0.12 vs. 1.52±0.13 g; n=10; P<0.01) with a 17% reduction in body weight. The subsequent growth pattern showed ‘catch-up growth’ in the UN group. Thus, although there was no difference in body weight at P14 in the UN group compared with the C group (7.5±0.4 vs. 6.8±0.3 g; n=10; P>0.1) (Fig. 1), offspring from the UN group showed a significant increase in body weight compared with the C group at P90 (UN, 28±1 g; C, 23±1.7 g; n=10; P<0.01) (Fig. 1). Weight was increased in the UN group (>20%) compared with the C group at P90.

### Food intake

**Figure 2:**
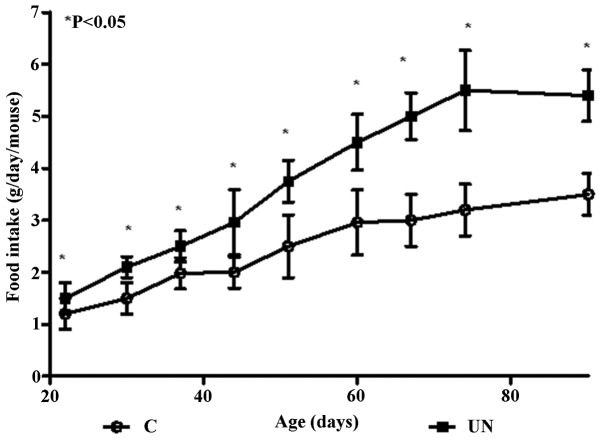
Postweaning food intake of male offspring between 21 and 90 days-old in C and prenatal UN groups (n=10 per group from 6 litters). ^*^P<0.05, vs. C. C, control; UN, undernutrition.

Food intake was monitored following weaning in the offspring from the UN group compared with the C group and increased food intake was noted in the UN group. In early postnatal life, the UN group continued with weight gain and this trend of hyperphagia persisted throughout adult life. Food intake was significantly increased in the UN group compared with the C group offspring in adulthood at P90 (P<0.05; Fig. 2).

### Ob-Rb leptin receptor expression

**Figure 3:**
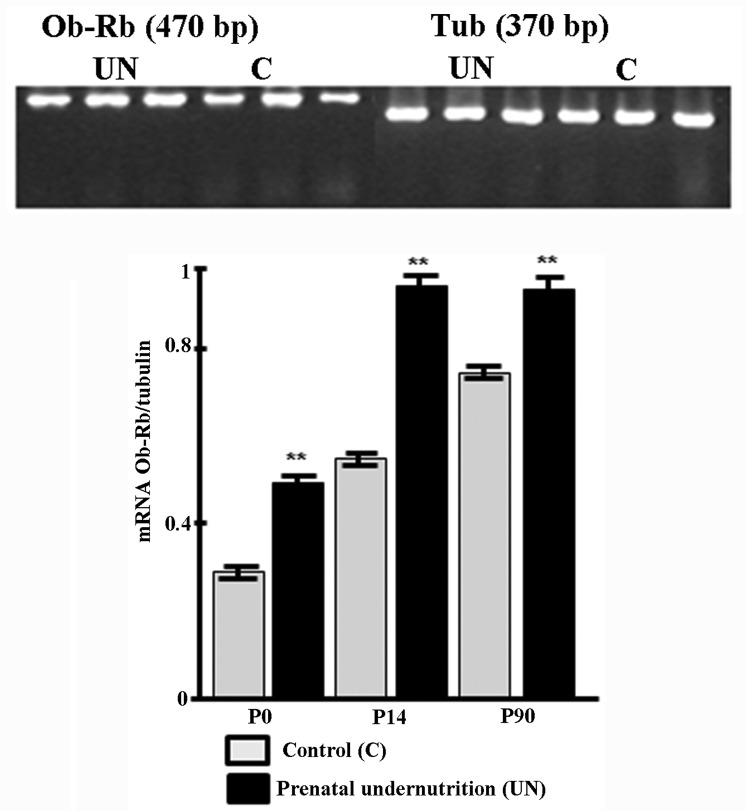
Changes in mRNA expression of Ob-Rb in the hypothalamus at postnatal days 0 (P0), 14 (P14) and 90 (P90) in UN and C groups and representation of Ob-Rb in 2% agarose gels in the presence of ethidium bromide. ^**^P<0.01, vs. C. C, control; UN, undernutrition; P, postnatal.

In the UN group, a significant increase in the Ob-Rb receptor expression was observed in the hypothalamus at P14 (P<0.01) and P90 (P<0.01) (Fig. 3).

### Effects of prenatal UN on 5-HT_1A_ and D1 and D2 receptor expression in the hypothalamus

**Figure 4:**
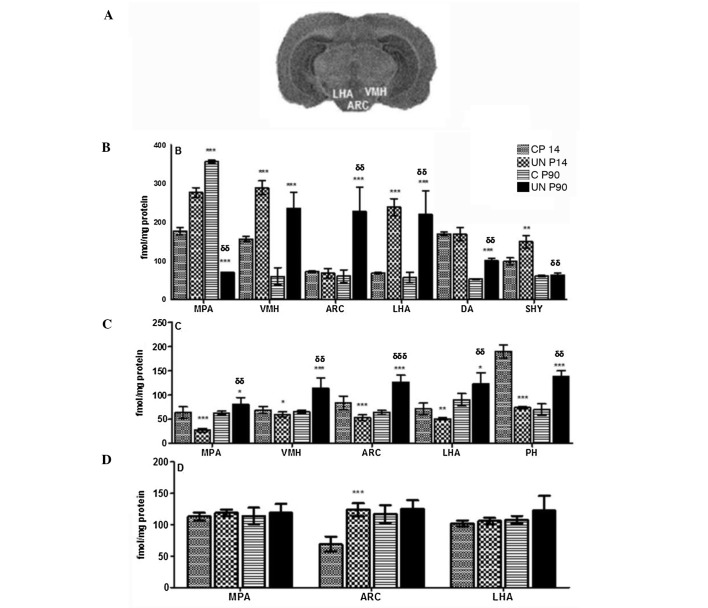
Autoradiographic distribution of 5. (A) Representation in brain of 5-HT1A; (B) 5-HT1A with [^3^H]8-OH-DPAT binding (fmol/mg protein); (C) distribution of D1 with [^3^H]SCH23390 binding (fmol/mg protein); and (D) distribution of D2 [^3^H]raclopride binding (fmol/mg protein) in the hypothalamus of mice. ^*^P<0.05, ^**^P<0.01 and ^***^P<0.001, UN vs. control; ^δ^P<0.05, ^δδ^P<0.01 and ^δδδ^P<0.001, UN P14 vs. UN P90 offspring. CP14, control; UN, undernutrition. P, postnatal; D1, dopamine 1; D2, dopamine 2.

Comparison of the UN group with the C group at P14 revealed that the 5-HT_1A_ receptor was increased in the ventromedial nucleus of the hypothalamus (VMH; +84%; 289±18 vs. 157±7; P<0.001), in the medial preoptic area (MPA; +56%; 277±2 vs. 177±10; P<0.001) and the lateral area of the hypothalamus (LHA; +251%; 239±22 vs. 68±2; P< 0.001). At P90, the UN group had an increase in the dorsal hypothalamic area (+64%; 87±13 vs. 53±9; P<0.01), VMH (+293%; 236±42 vs. 60±22; P<0.001), LHA (+279%; 220±26 vs. 58±13; P<0.001) and arcuate nucleus (ARC; +273%; 228±62 vs. 61±16; P<0.001) (Fig. 4A and B).

In the UN group at P14, a decrease in D1 receptor expression was observed in the MPA (−58%; 27±3 vs. 64±2; P<0.001), VMH (−13%; 60±1 vs. 69±3; P<0.05), ARC (−37%; 53±6 vs. 84±4; P<0.001), LHA (−42%; 41±1 vs. 71±2; P<0.001) and posterior hypothalamic area (−68%; 74±2 vs. 228±14; P<0.001) as compared with group C, however, there was an increase at P90 (Fig. 4C). By contrast, in the UN group at P14, there was an increase in D2 receptor expression in the ARC (+80%; 124±1 vs. 69±2; P<0.001), although no differences were observed at P90 (Fig. 4D).

## Discussion

Results of the current study present three novel observations. First, data demonstrate that the prenatal UN group negatively impacted development of the offspring (UN), however, following birth the rate and timing of postnatal catch-up growth played critical and significant roles. Second, accelerated body weight gain continued following weaning and was associated with altered anorexigenic regulatory mechanisms (leptin receptor). The data also show that developmental adaptation ensures fetal survival of the 5HT_1A_, D1 and D2 receptors in the hypothalamus. These observations emphasize the plasticity and potential of critical appetite-regulating neurotransmitters in the pathogenesis of fetal programming-induced obesity.

Fetal programming corresponded to an attempt of the fetus to adapt to the adverse conditions encountered *in utero* (9). These adaptations are likely to be beneficial if the conditions prevail later in life but become detrimental for normal or plentiful nutrition, favoring the appearance of obesity. Furthermore, the environment encountered during fetal life and infancy is significantly associated with the risk of diseases in adult life (16). Thus, UN during pregnancy is involved in the programming of offspring for the development of obesity and diabetes (17). To explain these causal relationships, it has been suggested that adaptations during the critical phases of growth and development may ensure the maintenance of homeostasis. Therefore, survival when the environment is compromised. Studies exploring the ‘thrifty phenotype’ hypothesis in animal models have indicated that long-term obesity and disease risk markers may be programmed by alterations in maternal nutrition, for example protein restriction (18–20) or by reduced nutritional supply to the fetus by uterine artery ligation in late pregnancy (18,19). In addition, maternal high-fat diet consumption during gestation, independent of obesity, increases the risk of offspring developing behavioral disorders, including anxiety.

In pregnant mice, production of serotonin and the expression of tryptophan hydroxylase 1 (Tph1), the rate-limiting enzyme in the synthesis of the 5HT pathway, were highly elevated in β-cells. Similar elevation in the expression of the Gαq protein coupled 5HT receptor gene, Htr2b, was also noted. Moreover, inhibition of Tph1 or Htr2b blocked normal increase of β-cell mass during pregnancy and resulted in glucose intolerance in mice (18,21).

Thus, current evidence has demonstrated that the ghrelin orexigenic effect is mediated by the selective modulation of hypothalamic fatty acid metabolism (18,22). Moreover, ob/ob mice exhibited reductions in food intake and body weight when treated with D1 and D2 agonists (18,23).

In the present study, exposure to maternal food restriction during gestation resulted in LBW offspring. However, with *ad libitum* feeding during early postnatal life, mice recovered their body weight by P14. In the hypothalami, the VMH, LHA and several other hypothalamic nuclei are well established as centers for metabolism regulation and have potent modulator effects on daily food intake mediated primarily via lower brainstem nuclei (18,24–26).

In the group of UN mice in the present study, food intake was increased and the group demonstrated hyperphagia. In addition, the 5-HT_1A_ receptor was elevated in VMH and LHA at P14 and P90, but was reduced in the ARC, indicating a decrease in 5-HT_1A_. This was directly associated with negative regulation of food intake, indicating hyperphagia associated with fetal programming. Previous studies have shown that agents that mimic or enhance 5-HT activity produced hypophagia (27) and weight loss, and inhibited neuropeptide Y (NPY) neuronal activity. In addition, drugs that block 5-HT release, stimulate feeding and NPY (28,29).

Results of this study suggest that 5-HT may tonically inhibit NPY neurons and mediate the effects of 5-HT serotonin on energy homeostasis in the ARC. The present study indicates that the 5-HT_1A_ receptor is likely to modify the regulation between the NPY and 5-HT system in the ARC during LBW, with an impact on adult life. Moreover, during neonatal development, food intake must be maximized to support growth, yet plasma leptin levels are relatively high. These high levels of leptin during the postnatal period have been reported in rats and mice. Similarly, Ob-Rb receptor expression was increased in the hypothalamus. Little is known about the co-regulation of Ob-R and Ob-Rb gene expression, receptor number or the impact of receptor regulation on leptin sensitivity (12). In this study, Ob-Rb receptor expression was increased with hyperphagia in mice with prenatal UN and an increase of food intake in early postnatal life.

Previous studies (30) have shown the importance of leptin and its association with dopamine in the modulation of food intake. In the present study, the D1 receptor was reduced in the hypothalamic nuclei in the UN group at P14. However, the D1 receptor at P90 was increased, which was important in ARC since these have been involved in metabolic changes. Thus, if dopaminergic receptor expression in the hypothalamus is controlled by dopamine release, it is possible that upregulation of the D1 receptor mRNA in the VMH and a decrease in the LHA of obese rats may be due to a low or high local dopamine concentration, respectively (31,32). In addition, alterations in D2 receptor levels were compared in Zucker obese (fa/fa) and lean (Fa/Fa) rats at 1 and 4 months of age, respectively, under two varying feeding conditions (restricted and unrestricted food access) using *in vivo* PET imaging and *in vitro* autoradiography. D2 receptors were higher at 1 than at 4 months of age and that food-restricted animals had higher D2 receptor levels than unrestricted animals (31,32). Thus, in the present study, increased D2 receptor expression in the ARC at P14 indicated a correlation with catch-up growth following prenatal UN. More studies are required to evaluate leptin effects on brain structure, function and metabolism, and concomitantly, to provide a solid foundation for studies aiming to assess possible roles of leptin, 5-HT and dopamine in food intake and in UN.

In conclusion, prenatal UN during gestation has defined time windows with long-term effects on weight gain and metabolism. In addition, overfeeding immediately following fetal growth retardation induces catch-up growth. Therefore, hyperphagia resulting from early programming indicated changes in the dopaminergic and serotonergic system that may program a state of obesity during adulthood.

## Figures and Tables

**Table I:** Incubation conditions for serotonergic and dopaminergic receptors.

Receptor	Refs	Ligand binding	Non-specific buffer	Incubation conditions	Pre-incubation buffer	Incubation	Washing	Exposure time, days
5-HT_1A_	14	2 nM [^3^H]8-OH-DPAT (specific activity 106 Ci/mmol)	10 μM WAY100635	0.17 M Tris-HCl, 4 mM CaCl_2_ and 0.01% ascorbic acid (pH 7.4)	30 min at 22°C	60 min at 22°C	2×5 min at 4°C	60
D1	15	2 nM [^3^H]SCH233902 (specific activity 85 Ci/mmol)	1 μM butaclamol	50 mM Tris HCl, 154 mM NaCl, 1 mM EDTA and 0.1% albumine (pH 7.4)	10 min at 22°C	90 min at 22°C and 30 nM ketanserine	2×5 min at 4°C	90
D2	13	0.55 nM [^3^H]raclopride (specific activity 60.1 Ci/mmol)	1 μM butaclamol	50 mM Tris HCl, 150 mM NaCl and 0.1% ascorbic acid (pH 7.4)	20 min at 22°C	45 min at 22°C	2×5 min at 4°C	90

D1, dopamine 1; D2, dopamine 2.
